# Ab Initio Calculations of Spin Waves: A Review of Theoretical Approaches and Applications

**DOI:** 10.3390/ma18184431

**Published:** 2025-09-22

**Authors:** Michael Neugum, Arno Schindlmayr

**Affiliations:** Department Physik, Universität Paderborn, 33095 Paderborn, Germany

**Keywords:** spin waves, magnons, many-body perturbation theory, time-dependent density-functional theory, adiabatic local-density approximation

## Abstract

Spin waves represent an important class of low-energy excitations in magnetic solids, which influence the thermodynamic properties and play a major role in technical applications, such as spintronics or magnetic data storage. Despite the enormous advances of ab initio simulations in materials science, quantitative calculations of spin-wave spectra still pose a significant challenge, because the collective nature of the spin dynamics requires an accurate treatment of the Coulomb interaction between the electrons. As a consequence, simple lattice models like the Heisenberg Hamiltonian are still widespread in practical investigations, but modern techniques like time-dependent density-functional theory or many-body perturbation theory also open a route to material-specific spin-wave calculations from first principles. Although both are in principle exact, actual implementations necessarily employ approximations for electronic exchange and correlation as well as additional numerical simplifications. In this review, we recapitulate the theoretical foundations of ab initio spin-wave calculations and analyze the common approximations that underlie present implementations. In addition, we survey the available results for spin-wave dispersions of various magnetic materials and compare the performance of different computational approaches. In this way, we provide an overview of the present state of the art and identify directions for further developments.

## 1. Introduction

Spin waves represent an important class of elementary excitations in magnetically ordered materials with major implications for both fundamental and applied science [1]. They originate from propagating disturbances of the aligned magnetic moments, usually the electron spins, which can be induced either by thermal excitation or by external fields. In the absence of a magnetic anisotropy, the spin waves exhibit no energy gap and therefore strongly influence the material properties even at a very low temperature *T*. In ferromagnets, for example, they cause a reduction in the magnetization proportional to $T^{3/2}$ in accordance with Bloch’s law [2] and give rise to a $T^{3/2}$ term in the specific heat capacity. Spin waves may also couple to charge excitations, thereby causing a significant renormalization of the quasiparticle energies and lifetimes near the Fermi level [3]. Another consequence of this coupling is a temperature-dependent contribution to the resistivity, which reflects the varying number of thermally excited spin waves, as well as a pronounced magnetoresistance effect [4]. The scattering due to spin waves furthermore limits the spin-dependent mean free path of hot electrons in magnetotransport experiments [5]. On a different note, spin waves have been proposed as a possible mediator for the electron-pairing mechanism in unconventional high-temperature superconductors [6]. This is supported by a growing body of experimental and theoretical evidence, not only for cuprates [7,8], but also for heavy-fermion systems like UGe_2_ or URhGe [9,10], which are remarkable for exhibiting superconductivity in the ferromagnetic phase.

From the perspective of technological applications, spin waves are at the heart of the rapidly evolving field of magnonics [11], which derives its name from the quantized quasiparticles of spin waves, magnons. Going beyond conventional spintronics, which also exploits the electron spin in order to store and process information, spin waves allow spin-encoded signal transmissions without the actual physical transport of charge carriers. As a consequence, magnonic devices avoid current-related Ohmic losses, such as Joule heating, and promise much higher clock frequencies combined with lower energy consumption. Although there are still many fundamental challenges [12], several devices, such as magnon transistors for all-magnon data processing [13], have already been demonstrated as functional prototypes.

For the proper understanding of spin-wave dynamics in real materials and the optimal design of technological devices, predictive numerical simulations are an essential tool. In the past, most computational studies were based on the classical Heisenberg model, using either empirical parameters or values derived from ab initio calculations [14], but the underlying assumption of localized magnetic moments with constant magnitude is often inappropriate, especially for itinerant electron systems, and the various possible methods of mapping to a discrete lattice model and determining the exchange parameters create substantial ambiguity [15,16,17,18,19,20]. Another common technique, the frozen-magnon method, employs constrained ground-state total-energy calculations for noncollinear spin-spiral configurations with finite wave vectors to estimate the spin-wave dispersion [21,22,23,24]. In this way, it avoids the mapping problem, but it ignores the dynamic nature of spin-wave excitations, which leads to systematic errors. Additionally, it cannot describe the finite lifetime of magnons or single-particle Stoner excitations that coexist with collective spin-wave modes, and fails in the case of multiple spin-wave branches with the same wave vector, which are known to occur even in apparently simple systems like elementary nickel [25]. Although such schemes may be classified as ab initio in the sense that they contain no empirical parameters, they are not covered in this review due to their inherent limitations.

In contrast, many-body perturbation theory (MBPT) and time-dependent density-functional theory (TDDFT) are designed to enable an exact description of spin excitations from first principles. Both center on the transverse dynamic spin susceptibility, whose poles as a function of frequency correspond directly to the complete energy spectrum of spin-wave and Stoner excitations, which are treated on the same footing. Although both approaches are in principle exact and take dynamic effects fully into account, their theoretical foundations are very different: The former relies on Green functions and includes the Coulomb interaction between excited electrons and holes explicitly through a summation of Feynman diagrams [26,27]. The latter is based on the Runge–Gross theorem [28] and derives the susceptibility from the response of the spin density to a time-dependent external magnetic field, where the Coulomb interaction is contained in implicit density functionals. As a consequence, approximations for electronic exchange and correlation, which are necessary in practical applications, deviate considerably in the two frameworks. While a number of independent implementations have been reported, all are subject to a variety of additional numerical simplifications and cutoffs, such as basis-set truncation, introduced to reduce the high computational expense. Due to a lack of accurate theoretical benchmark data, the quantitative errors resulting from the individual approximations are not always transparent, and a direct comparison with other ab initio calculations from the literature is rare.

One aim of this review is to collect pertinent spin-wave results from the scientific literature that were generated independently with distinct implementations, using a variety of basis sets, computational strategies, and additional numerical simplifications, and to compare these to each other as well as to available experimental measurements. In this way, we elucidate the accuracy and general characteristics of both schemes independent of the numerical details of specific codes.

In order to clarify the present state of the art in this field and make it accessible to a wider community, we review the theoretical foundations of MBPT and TDDFT within a common notational framework in Section 2 and highlight the distinct approximations for the transverse dynamic spin susceptibility that underlie common implementations. In Section 3, we then provide an overview of material-specific calculations reported in the scientific literature until now and compare the resulting spin-wave dispersions from different methods with experimental measurements for several selected ferromagnets. This survey not only allows a systematic quantitative assessment of the various computational approaches but also illustrates the range of applications that are presently within reach. Finally, we summarize our conclusions in Section 4 and point out directions for further research. To keep the formulas simple, we use Hartree atomic units except where explicitly stated otherwise.

## 2. Theoretical Background

The central quantity for the ab initio investigation of spin excitations is the dynamical spin susceptibility, which can be identified with the retarded spin–spin correlation function

(1) $$\chi^{ij}\left(r,r^{\prime };t-t^{\prime }\right)=-i\langle 0|\left[{\hat{S}}_{i}\left(r,t\right),{\hat{S}}_{j}\left(r^{\prime },t^{\prime }\right)\right]|0\rangle \Theta \left(t-t^{\prime }\right),$$

where |0〉 represents the stationary ground state of a many-electron system with the energy $E_{0}$ in the absence of external fields, and ${\hat{S}}_{i}\left(r,t\right)$ with i∈{x,y,z} is a Cartesian vector component of the time-dependent spin-density operator in the Heisenberg picture [27]. It depends only on the difference $t-t^{\prime }$, because the Hamiltonian of the unperturbed system is time-invariant. The square brackets denote the commutator of two quantum-mechanical operators. In the case of collinear systems, where the spin density is oriented along one global magnetization axis, here chosen as the *z* direction, it is sufficient to consider only the so-called transverse part of the spin susceptibility, which is defined as

(2) $$\chi^{+-}\left(r,r^{\prime };t-t^{\prime }\right)=-i\langle 0|\left[{\hat{S}}_{+}\left(r,t\right),{\hat{S}}_{-}\left(r^{\prime },t^{\prime }\right)\right]|0\rangle \Theta \left(t-t^{\prime }\right)$$

in terms of the ladder operators ${\hat{S}}_{\pm }={\hat{S}}_{x}\pm i{\hat{S}}_{y}$ that raise or lower the total spin by one, instead of the full tensor quantity. This scalar function corresponds to a linear combination of the Cartesian components, which can be further simplified to

(3) $$\chi^{+-}\left(r,r^{\prime };t-t^{\prime }\right)=2\chi^{xx}\left(r,r^{\prime };t-t^{\prime }\right)-2i\chi^{xy}\left(r,r^{\prime };t-t^{\prime }\right)$$

if rotational symmetries around the *z* axis in spin space that apply to collinear magnetic systems without spin–orbit coupling are exploited. In the next step, we insert a complete set of eigenstates $|n,S_{z}\pm 1\rangle$, whose total spin differs from the ground-state value $S_{z}$ by 1, with associated eigenvalues $E_{n,S_{z}\pm 1}$. After a Fourier transformation from the time domain to frequency space, we thus obtain the spectral representation [29]

(4) $$\chi^{+-}\left(r,r^{\prime };\omega \right)=\lim_{\eta \to 0}\left(\sum_{n}\frac{\langle 0|{\hat{S}}_{+}\left(r\right)|n,S_{z}-1\rangle \langle n,S_{z}-1|{\hat{S}}_{-}\left(r^{\prime })\right|0\rangle }{\omega -(E_{n,S_{z}-1}-E_{0})+i\eta }-\sum_{n}\frac{\langle 0|{\hat{S}}_{-}\left(r^{\prime }\right)|n,S_{z}+1\rangle \langle n,S_{z}+1|{\hat{S}}_{+}\left(r\right)|0\rangle }{\omega +(E_{n,S_{z}+1}-E_{0})+i\eta }\right),$$

where the infinitesimal η>0 arises from an exponential convergence factor that must be introduced to ensure that the integral over the time axis is well defined. From this result, one can see that the poles of the transverse dynamic spin susceptibility in frequency space correspond to the exact transition energies between the ground state and excited states with a relative change in total spin by ±1 and identical particle number. These comprise single-particle spin flips, known as Stoner excitations, that can be interpreted as transitions from occupied states in the majority or minority band to unoccupied states in the opposite spin channel, as well as collective spin-wave excitations, also called magnons. Despite their very different physical nature, the two are thus treated on the same footing. By projecting the susceptibility onto plane waves with a wave vector q in the first Brillouin zone, one eventually obtains the dispersion relation ω(q) of the excitation modes.

In principle, the excitation energies obtained from the transverse spin susceptibility include the full strength of electronic exchange and correlation, but the restriction to a single spin flip implies a description of the spin waves as independent quasiparticles. This is appropriate for experimental situations where the density of spin waves is sufficiently low so that magnon–magnon scattering can be neglected, for example by thermal excitation at room temperature. Additionally, magnon–phonon scattering is also ignored because of the underlying Born–Oppenheimer approximation. Of course, both may later be incorporated in a perturbative manner, if desired.

Finally, the fluctuation–dissipation theorem asserts that the dynamic spin susceptibility, originally defined as a spin–spin correlation function in Equation (1) above, is also identical to the linear-response function that describes the change in the magnetization density

(5) $$m\left(r,t\right)=m^{\left(0\right)}\left(r\right)-\int \chi \left(r,r^{\prime };t-t^{\prime }\right)B_{\mathrm{ext}}^{\left(1\right)}\left(r^{\prime },t^{\prime }\right)\;d^{d}r^{\prime }\;dt^{\prime }+\ldots$$

due to a time-dependent external magnetic field $B_{\mathrm{ext}}^{\left(1\right)}\left(r^{\prime },t^{\prime }\right)$ that couples to the electron spins. The former is defined as $m\left(r,t\right)=\gamma_{e}S\left(r,t\right)$ with the gyromagnetic ratio $\gamma_{e}=-1$ in atomic units, and the Heaviside step function $\Theta (t-t^{\prime })$ in the retarded correlation function ensures causality. As a consequence, it may be obtained from the functional derivative

(6) $${\left.\chi \left(r,r^{\prime };t-t^{\prime }\right)=-\frac{\delta m(r,t)}{\delta B_{\mathrm{ext}}\left(r^{\prime },t^{\prime }\right)}\right|}_{B_{\mathrm{ext}}\left(r^{\prime },t^{\prime }\right)=B_{\mathrm{ext}}^{\left(0\right)}\left(r^{\prime }\right)}.$$

This opens a pathway for the practical computation of the transverse spin susceptibility using any ab initio method that is capable of accurately predicting physical observables of interacting many-electron systems under time-dependent external fields. Foremost among these are time-dependent density-functional theory and many-body perturbation theory. Both of these approaches have been successfully demonstrated for spin-wave calculations in actual implementations and are discussed in the following.

### 2.1. Time-Dependent Density-Functional Theory

The complexity of the many-body problem lies in the Coulomb interaction of the electrons. The basic idea of the Kohn–Sham formalism within density-functional theory (DFT) is to replace the explicit pair interaction by an effective one-particle potential $V_{\mathrm{eff}}^{\left(0\right)}\left(r\right)$ and magnetic field $B_{\mathrm{eff}}^{\left(0\right)}\left(r\right)$ constructed in such a way that they reproduce the exact ground-state electron distribution and magnetization density of the actual physical system. This can be generalized to time-dependent situations, where the variation of the magnetization density may be expanded as

(7) $$m\left(r,t\right)=m^{\left(0\right)}\left(r\right)-\int \chi_{0}\left(r,t,r^{\prime },t^{\prime }\right)B_{\mathrm{eff}}^{\left(1\right)}\left(r^{\prime },t^{\prime }\right)\;d^{d}r^{\prime }\;dt^{\prime }+\ldots$$

up to the first order in the additional time-dependent part of the effective field $B_{\mathrm{eff}}^{\left(1\right)}$. The spin susceptibility of this auxiliary electron system is hence given by

(8) $${\left.\chi_{0}\left(r,r^{\prime },t-t^{\prime }\right)=-\frac{\delta m(r,t)}{\delta B_{\mathrm{eff}}\left(r^{\prime },t^{\prime }\right)}\right|}_{B_{\mathrm{eff}}\left(r^{\prime },t^{\prime }\right)=B_{\mathrm{eff}}^{\left(0\right)}\left(r^{\prime }\right)}$$

and is commonly referred to as the Kohn–Sham or noninteracting spin susceptibility. A comparison with Equation (5) immediately confirms the identity

(9) $$-\int \chi \left(r,r^{\prime };t-t^{\prime }\right)B_{\mathrm{ext}}^{\left(1\right)}\left(r^{\prime },t^{\prime }\right)\;d^{d}r\;dt^{\prime }=m^{\left(1\right)}\left(r,t\right)=-\int \chi_{0}\left(r,r^{\prime };t-t^{\prime }\right)B_{\mathrm{eff}}^{\left(1\right)}\left(r^{\prime },t^{\prime }\right)\;d^{d}r\;dt^{\prime }.$$

Next, the effective magnetic field is written as the sum

(10) $$B_{\mathrm{eff}}^{\left(1\right)}\left(r^{\prime },t^{\prime }\right)=B_{\mathrm{ext}}^{\left(1\right)}\left(r^{\prime },t^{\prime }\right)+B_{\mathrm{xc}}^{\left(1\right)}\left(r^{\prime },t^{\prime }\right)$$

of the applied external field and an additional exchange-correlation contribution. By virtue of the Runge–Gross theorem [28], the latter is a unique functional of the electron density and the magnetization density. Therefore, its linear variation due to a change in the magnetization density is given by

(11) $$B_{\mathrm{xc}}^{\left(1\right)}\left(r^{\prime },t^{\prime }\right)=-\int f_{\mathrm{xc}}\left(r^{\prime },r^{\prime \prime };t^{\prime }-t^{\prime \prime }\right)m^{\left(1\right)}\left(r^{\prime \prime },t^{\prime \prime }\right)\;d^{d}r^{\prime \prime }\;dt^{\prime \prime }+\ldots ,$$

where the linear-response function

(12) $$f_{\mathrm{xc}}\left(r^{\prime },r^{\prime \prime };t^{\prime }-t^{\prime \prime }\right)=-{\left.\frac{\delta B_{\mathrm{xc}}\left(r^{\prime },t^{\prime }\right)}{\delta m(r^{\prime \prime },t^{\prime \prime })}\right|}_{m\left(r^{\prime \prime },t^{\prime \prime }\right)=m^{\left(0\right)}\left(r^{\prime \prime }\right)}$$

is known as the exchange-correlation kernel. After inserting the left-hand side of Equation (9) into $m^{\left(1\right)}\left(r^{\prime \prime },t^{\prime \prime }\right)$ and collecting all terms linear in $B_{\mathrm{ext}}^{\left(1\right)}$, the spin susceptibility is thus seen to obey the Dyson-type integral equation

(13) $$\chi \left(r,r^{\prime };\omega \right)=\chi_{0}\left(r,r^{\prime };\omega \right)+\int \chi_{0}\left(r,r^{\prime \prime };\omega \right)f_{\mathrm{xc}}\left(r^{\prime \prime },r^{\prime \prime \prime };\omega \right)\chi \left(r^{\prime \prime \prime },r^{\prime };\omega \right)\;d^{d}r^{\prime \prime }\;d^{d}r^{\prime \prime \prime },$$

where the Fourier transformation to frequency space was used to convert the convolution integrals in the time domain into simple products of the Fourier coefficients.

Since the spin susceptibility is a tensor, the multiplications in Equation (13) should, in general, be interpreted as matrix multiplications. For collinear magnetic systems, it becomes block diagonal, however, because the linear variation of the magnetization along the *z* direction decouples from the projection in the xy plane [30]. Furthermore, the nondiagonal elements of the exchange-correlation kernel vanish. In such situations, it is possible to relate the relevant transverse parts through an equivalent scalar equation of the form

(14) $$\chi^{+-}\left(r,r^{\prime };\omega \right)=\chi_{0}^{+-}\left(r,r^{\prime };\omega \right)+\int \chi_{0}^{+-}\left(r,r^{\prime \prime };\omega \right)f_{\mathrm{xc}}^{+-}\left(r^{\prime \prime },r^{\prime \prime \prime };\omega \right)\chi^{+-}\left(r^{\prime \prime \prime },r^{\prime };\omega \right)\;d^{d}r^{\prime \prime }\;d^{d}r^{\prime \prime \prime },$$

where the effective transverse exchange-correlation kernel follows from the nonzero diagonal elements of the full tensor according to

(15) $$f_{\mathrm{xc}}^{+-}\left(r^{\prime \prime },r^{\prime \prime \prime };\omega \right)=\frac{1}{2}f_{\mathrm{xc}}^{xx}\left(r^{\prime \prime },r^{\prime \prime \prime };\omega \right).$$

For practical purposes, the susceptibilities and the kernel are usually projected onto a basis of orthonormal Bloch functions with wave vectors q, such as plane waves. In this case, Equation (14) transforms into a matrix equation

(16) $$\chi^{+-}\left(q,\omega \right)=\chi_{0}^{+-}\left(q,\omega \right)+\chi_{0}^{+-}\left(q,\omega \right)f_{\mathrm{xc}}^{+-}\left(q,\omega \right)\chi^{+-}\left(q,\omega \right),$$

whose solution is then obtained by means of a matrix inversion as

(17) $$\chi^{+-}\left(q,\omega \right)={\left[1-\chi_{0}^{+-}\left(q,\omega \right)f_{\mathrm{xc}}^{+-}\left(q,\omega \right)\right]}^{-1}\chi_{0}^{+-}\left(q,\omega \right).$$

In most actual implementations of TDDFT for magnetic excitations, one begins by performing a self-consistent ground-state calculation within spin-DFT. For collinear systems, this involves separate Kohn–Sham equations for the two spin channels

(18) $$\left[-\frac{1}{2}\nabla^{2}+V_{\mathrm{eff}}\left(r\right)+\frac{1}{2}\sigma_{\alpha \alpha }^{z}B_{\mathrm{eff}}^{\left(0\right)}\left(r\right)\right]\varphi_{n\alpha }\left(r\right)=\epsilon_{n\alpha }\varphi_{n\alpha }\left(r\right)$$

with α∈{↑,↓}. The sign factors $\sigma_{↑↑}^{z}=+1$ and $\sigma_{↓↓}^{z}=-1$ are the diagonal elements of the third Pauli matrix. From the calculated eigenfunctions and eigenvalues, the Kohn–Sham transverse spin susceptibility can then be constructed according to [31,32,33]

(19) $$\chi_{0}^{+-}\left(r,r^{\prime };\omega \right)=\lim_{\eta \to 0}\sum_{n,n^{\prime }}\frac{f\left(\epsilon_{n↑}\right)-f\left(\epsilon_{n^{\prime }↓}\right)}{\omega -(\epsilon_{n^{\prime }↓}-\epsilon_{n↑})+i\eta }\varphi_{n↑}^{*}\left(r\right)\varphi_{n^{\prime }↓}\left(r\right)\varphi_{n^{\prime }↓}^{*}\left(r^{\prime }\right)\varphi_{n↑}\left(r^{\prime }\right),$$

where f(ϵ) denotes the Fermi occupation numbers, which reduce to a step function at zero temperature. The number of unoccupied states included in the spectral summation must be finite in practice but can be increased to yield well-converged results. The precise number depends on the material and on the energy window in question. Obviously, all transition energies $|\epsilon_{n^{\prime }↓}-\epsilon_{n↑}|$ inside the relevant range of ω must be included, but transitions at higher energies still influence the low-energy part of $\chi_{0}^{+-}\left(r,r^{\prime };\omega \right)$ due to the slow decay of the Lorentzian lineshape functions. Spin-wave calculations for elemental transition metals like nickel, which has five valence bands resulting from the occupied 3*d* and 4*s* orbitals, typically use about 20 unoccupied conduction bands [29,33] for good convergence.

In principle, the Fermi occupation numbers in Equation (19) could also be used to simulate different temperature regimes. As in static self-consistent DFT calculations, it is indeed common to choose a finite smearing instead of a step function to enhance the convergence behavior, especially in calculations for metallic systems. However, this should be regarded purely as a numerical convergence technique, because other important effects of temperature on the spin-wave dispersion, such as the thermal expansion of the crystal lattice or magnon–phonon coupling, are still neglected. The results from theoretical simulations thus correspond best to experimental measurements at low temperature.

Alternatively, the computationally expensive summation over all unoccupied states in Equation (19) can be avoided by using numerically constructed Green functions [30] or by calculating the modification for the wave functions of the occupied eigenstates due to the external magnetic field explicitly within time-dependent density-functional perturbation theory. In the latter case, the resulting self-consistent Sternheimer equation for the linear variation of the wave functions may either be solved directly [34,35] or by means of the Liouville–Lanczos scheme [36]. It is also possible to determine the response of the magnetization density from a real-time propagation [37,38,39]. All of these approaches are, in principle, mathematically equivalent, but the different implementations necessarily lead to distinct convergence parameters and numerical approximations.

Although the exact mathematical expressions for the effective potential and magnetic field in Equation (18) are unknown, modern parametrizations for the exchange-correlation functional, such as the local-density approximation (LDA) or generalized gradient approximations (GGAs), ensure a highly accurate description of the eigenfunctions and eigenvalues in most cases. Therefore, the Kohn–Sham susceptibility is not considered as a major source of errors in typical material-specific applications.

Finally, although the exchange-correlation kernel defined in Equation (12) is a well-defined quantity in the context of TDDFT, no simple mathematical expressions that take the spatial and temporal nonlocality properly into account are currently available. In principle, Equation (12) could be evaluated with any approximation for the exchange-correlation energy functional by exploiting the adiabatic relation

(20) $$B_{\mathrm{xc}}\left(r^{\prime },t^{\prime }\right)=-{\left.\frac{\delta E_{\mathrm{xc}}\left[n,m\right]}{\delta m(r)}\right|}_{m(r)=m(r,t)},$$

but care must be taken to ensure consistency with the exchange-correlation potential and magnetic field in Kohn–Sham Equation (18) for the self-consistent ground-state calculation, which should be derived from the same functional. The most common approach is the adiabatic local-density approximation (ALDA), which simply employs the standard LDA functional from static spin-DFT

(21) $$E_{\mathrm{xc}}^{\mathrm{ALDA}}\left[n,m\right]=\int e_{\mathrm{xc}}\left(n(r),|m(r)|\right)n\left(r\right)\;d^{d}r,$$

where $e_{\mathrm{xc}}\left(n,m\right)$ denotes the exchange-correlation energy per particle for a spin-polarized homogeneous electron gas with density *n* and magnetization *m*, evaluated at the time-dependent electron and magnetization density. Although the LDA itself describes the spatial modulation of the stationary exchange-correlation potential $V_{\mathrm{xc}}^{\mathrm{ALDA}}\left(r\right)$ reasonably well, the resulting adiabatic kernel

(22) $$f_{\mathrm{xc},\mathrm{ALDA}}^{+-}\left(r,r^{\prime };t-t^{\prime }\right)=\frac{1}{2}{\left.\frac{\partial e_{\mathrm{xc}}\left(n,m\right)}{\partial m}\frac{n}{m}\right|}_{n=n^{\left(0\right)}\left(r\right),m=\left|m^{\left(0\right)}\left(r\right)\right|}\delta \left(r-r^{\prime }\right)\delta \left(t-t^{\prime }\right),$$

disregards essential nonlocal and dynamic effects. The most obvious consequence is that its Fourier transform $f_{\mathrm{xc},\mathrm{ALDA}}$ is neither wave-vector- nor frequency-dependent, unlike the exact kernel $f_{\mathrm{xc}}\left(q,\omega \right)$. As it equals the static long-wavelength limit $f_{\mathrm{xc},\mathrm{ALDA}}=\lim_{q\to 0}f_{\mathrm{xc}}\left(q,0\right)$, the ALDA is expected to perform best near the Γ point, where the magnon energies are small, and become increasingly inaccurate towards the edges of the Brillouin zone.

A possible alternative to the ALDA is the adiabatic generalized gradient approximation (AGGA), where the exchange-correlation energy functional depends not only on the local electron and magnetization density but also on their gradients. Although GGAs are often considered superior to the LDA in static ground-state calculations, the corresponding kernel $f_{\mathrm{xc},\mathrm{AGGA}}\left(q\right)$ is still frequency independent and ignores dynamic effects due to the adiabatic construction, while its ${\left|q\right|}^{2}$ behavior gives a poor account of the actual wave-vector dependence throughout the Brillouin zone [32]. Therefore, it is not a priori clear that gradient corrections in the AGGA will also improve the description of spin-wave dispersions. Due to these deficiencies, it is generally agreed that the approximation of the exchange-correlation kernel is the dominant source of errors in linear-response TDDFT.

### 2.2. Many-Body Perturbation Theory

Unlike TDDFT, which is based on the electron density and the magnetization density vector, many-body perturbation theory employs the spin-dependent Green function $G_{\alpha \beta }\left(r_{1},r_{2};t_{1}-t_{2}\right)$, whose components are labeled by α,β∈{↑,↓}. Formally defined as the time-ordered correlation function of a particle annihilation operator with spin α at $\left(r_{1},t_{1}\right)$ and a creation operator with spin β at $\left(r_{2},t_{2}\right)$ analogous to Equation (1), it describes the propagation of electrons for $t_{1}>t_{2}$ or holes for $t_{2}>t_{1}$ with full account of scattering by the ionic cores and the interelectronic Coulomb repulsion. As the spin is not conserved in the presence of spin–orbit coupling or a nonuniform magnetic field whose direction varies at different points in space, there is a finite probability for a spin flip during the propagation in such cases, which leads to nonzero off-diagonal elements of the Green function for general noncollinear magnetic systems. For brevity, we use the short-hand notation $G_{\alpha \beta }\left(12\right)$ in the following, where $\left(1\right)=\left(r_{1},t_{1}\right)$ denotes a set of position and time coordinates. Where necessary, $\left(1^{+}\right)$ stands for $\left(r_{1},t_{1}+\eta \right)$ with an infinitesimal η>0 to ensure the correct time ordering. The Green function obeys the equation of motion [40]

(23) $$\left(i\frac{\partial }{\partial t_{1}}+\frac{1}{2}\nabla_{r_{1}}^{2}-V_{\mathrm{ext}}\left(1\right)\right)G_{\alpha \beta }\left(12\right)+\sum_{\gamma }\sum_{i}m_{\alpha \gamma }^{i}B_{\mathrm{ext}}^{i}\left(1\right)G_{\gamma \beta }\left(12\right)-\sum_{\gamma }\int M_{\alpha \gamma }\left(13\right)G_{\gamma \beta }\left(32\right)\;d3=\delta \left(12\right)\delta_{\alpha \beta }$$

with i∈{x,y,z} and the magnetization operator $m^{i}=\gamma_{e}\sigma^{i}/2$, where $\sigma^{i}$ denotes the Pauli matrices. The equation of motion is equivalent to the Dyson equation: The first two terms on the left-hand side describe the propagation of an independent particle in the presence of an external scalar potential $V_{\mathrm{ext}}\left(1\right)$ and a magnetic field $B_{\mathrm{ext}}\left(1\right)$, whereas the third term describes all additional scattering events arising from the Coulomb interaction between the electrons by means of a nonlocal and time-dependent potential. The mass operator

(24) $$M_{\alpha \gamma }\left(13\right)=V_{H}\left(1\right)\delta \left(13\right)\delta_{\alpha \gamma }+\Sigma_{\alpha \gamma }\left(13\right)$$

comprises the classical local Hartree potential

(25) $$V_{H}\left(1\right)=\int v\left(12\right)n\left(2\right)d2=-i\sum_{\alpha }\int v\left(12\right)G_{\alpha \alpha }\left(22^{+}\right)d2$$

with the instantaneous Coulomb interaction $v\left(12\right)=v\left(r_{1}-r_{2}\right)\delta \left(t_{1}-t_{2}\right)$ and the self-energy $\Sigma_{\alpha \gamma }\left(13\right)$, which incorporates all additional quantum-mechanical exchange and correlation effects. Like the Hartree potential, it is itself a functional of the Green function, which can be formally expanded into an infinite series of Feynman diagrams.

Although Green functions are often used to study quasiparticle excitations as probed in photoemission spectroscopy, they also yield ground-state expectation values of physical observables. In contrast to DFT, where the Hohenberg–Kohn theorem merely establishes the existence of an appropriate density functional, the explicit mathematical form as a functional of the Green function [40]

(26) $$O=-i\sum_{\alpha ,\beta }\int \lim_{t_{3}\to t_{2}^{+}}{\left[O_{\beta \alpha }\left(r_{2}\right)G_{\alpha \beta }\left(23\right)\right]}_{r_{3}=r_{2}}d^{d}r_{2}$$

for a quantum-mechanical operator $O_{\beta \alpha }\left(r_{2}\right)$ in MBPT is known exactly. In particular, the elements of the magnetization density vector with $O_{\beta \alpha }\left(r_{2}\right)=m_{\beta \alpha }^{i}\delta \left(r_{1}-r_{2}\right)$ are given by

(27) $$m^{i}\left(1\right)=-i\sum_{\alpha ,\beta }m_{\beta \alpha }^{i}G_{\alpha \beta }\left(11^{+}\right),$$

from which the dynamic spin susceptibility

(28) $$\chi^{ij}\left(12\right)=-{\left.\frac{\delta m^{i}\left(1\right)}{\delta B_{\mathrm{ext}}^{j}\left(2\right)}\right|}_{B_{\mathrm{ext}}\left(2\right)=B_{\mathrm{ext}}^{\left(0\right)}\left(2\right)}=i\sum_{\alpha ,\beta }{\left.m_{\beta \alpha }^{i}\frac{\delta G_{\alpha \beta }\left(11^{+}\right)}{\delta B_{\mathrm{ext}}^{j}\left(2\right)}\right|}_{B_{\mathrm{ext}}\left(2\right)=B_{\mathrm{ext}}^{\left(0\right)}\left(2\right)}$$

may thus be obtained. The functional derivative is most easily calculated using the identity

(29) $$\frac{\delta G_{\alpha \beta }\left(11^{+}\right)}{\delta B_{\mathrm{ext}}^{j}\left(2\right)}=-\sum_{\gamma ,\delta }\int \int G_{\alpha \gamma }\left(13\right)\frac{\delta G_{\gamma \delta }^{-1}\left(34\right)}{\delta B_{\mathrm{ext}}^{j}\left(2\right)}G_{\delta \beta }\left(41^{+}\right)\;d3\;d4.$$

The inverse of the Green function follows from Equation (23) and equals

(30) $$G_{\gamma \delta }^{-1}\left(34\right)=\left[\left(i\frac{\partial }{\partial t_{3}}+\frac{1}{2}\nabla_{r_{3}}^{2}-V_{\mathrm{ext}}\left(3\right)\right)\delta_{\gamma \delta }+\sum_{i}m_{\gamma \delta }^{i}B_{\mathrm{ext}}^{i}\left(3\right)\right]\delta \left(34\right)-M_{\gamma \delta }\left(34\right).$$

Its derivative with respect to the external magnetic field is hence given by

(31) $$\frac{\delta G_{\gamma \delta }^{-1}\left(34\right)}{\delta B_{\mathrm{ext}}^{j}\left(2\right)}=m_{\gamma \delta }^{j}\delta \left(32\right)\delta \left(34\right)-\frac{\delta M_{\gamma \delta }\left(34\right)}{\delta B_{\mathrm{ext}}^{j}\left(2\right)}.$$

The remaining task is the derivative of the mass operator. The first contribution, the classical Hartree potential, is defined in Equation (25) and yields

(32) $$\frac{\delta V_{H}\left(3\right)}{\delta B_{\mathrm{ext}}^{j}\left(2\right)}=-i\sum_{\alpha }\int v\left(31\right)\frac{\delta G_{\alpha \alpha }\left(11^{+}\right)}{\delta B_{\mathrm{ext}}^{j}\left(2\right)}\;d1,$$

where the derivative of the Green function is the same as in Equation (29). In contrast, the exact mathematical form of the exchange-correlation self-energy is unknown. However, we can employ the chain rule for functional derivatives to express

(33) $$\frac{\delta \Sigma_{\gamma \delta }\left(34\right)}{\delta B_{\mathrm{ext}}^{j}\left(2\right)}=\sum_{\alpha ,\beta }\int \int I_{\gamma \delta ,\alpha \beta }\left(34,56\right)\frac{\delta G_{\alpha \beta }\left(56\right)}{\delta B_{\mathrm{ext}}^{j}\left(2\right)}\;d5\;d6$$

in terms of the derivative of the Green function and the interaction kernel

(34) $$I_{\gamma \delta ,\alpha \beta }\left(34,56\right)=\frac{\delta \Sigma_{\gamma \delta }\left(34\right)}{\delta G_{\alpha \beta }\left(56\right)}.$$

Like the exchange-correlation potential in density-functional theory, the unknown self-energy must be replaced by a tractable explicit formula in practical applications. The standard choice is the so-called GW approximation

(35) $$\Sigma_{\alpha \beta }\left(12\right)=iG_{\alpha \beta }\left(12\right)W\left(12\right),$$

which models the self-energy as the product of the Green function and the dynamically screened Coulomb interaction, which is spin-independent and satisfies the Dyson-type equation

(36) W(12)=v(12)+∫∫v(13)P(34)W(42) d3 d4.

It is usually constructed with the polarization function in the random-phase approximation

(37) $$P\left(34\right)=-i\sum_{\alpha ,\beta }G_{\alpha \beta }\left(34\right)G_{\beta \alpha }\left(43^{+}\right).$$

The GW approximation is known to yield accurate quasiparticle band structures for a wide range of crystalline materials [41], including transition metals and their compounds with magnetic ordering. The corresponding interaction kernel takes the form

(38) $$I_{\alpha \beta ,\gamma \delta }\left(12,34\right)=iW\left(12\right)\delta \left(13\right)\delta \left(24\right)\delta_{\alpha \gamma }\delta_{\beta \delta }+iG_{\alpha \beta }\left(12\right)\frac{\delta W(12)}{\delta G_{\gamma \delta }\left(34\right)}.$$

In contrast to general noncollinear systems, where the eigenstates are two-component spinors with a position-dependent mixing of both spin-channels, all stationary eigenstates of collinear magnetic systems are pure spin-up or spin-down states. Under these circumstances, the Green function is diagonal in the spin components and can hence be written as $G_{\alpha \beta }\left(12\right)=G_{\alpha }\left(12\right)\delta_{\alpha \beta }$. Furthermore, the derivatives of the Hartree potential in Equation (32) and of the dynamically screened Coulomb interaction in Equation (38) do not contribute due to the orthogonality of the two spin channels. As a consequence, the transverse dynamic spin susceptibility can be simplified to

(39) $$\chi^{+-}\left(12\right)=-i\int \int G_{↓}\left(13\right)G_{↑}\left(41^{+}\right)\Gamma_{↓↑}\left(34,2\right)\;d3\;d4.$$

The vertex function is formally defined as

(40) $$\Gamma_{↓↑}\left(34,2\right)=-{\left[\frac{\delta G_{↓↑}^{-1}\left(34\right)}{\delta B_{\mathrm{ext}}^{x}\left(2\right)}-i\frac{\delta G_{↓↑}^{-1}\left(34\right)}{\delta B_{\mathrm{ext}}^{y}\left(2\right)}\right]}_{B_{\mathrm{ext}}\left(2\right)=B_{\mathrm{ext}}^{\left(0\right)}\left(2\right)}$$

and may be calculated from the integral equation

(41) $$\Gamma_{↓↑}\left(34,2\right)=\delta \left(32\right)\delta \left(42\right)+iW\left(34\right)\int \int G_{↓}\left(35\right)G_{↑}\left(64\right)\Gamma_{↓↑}\left(56,2\right)\;d5\;d6.$$

Physically, it describes spin-flip excitations in the form of electron–hole pairs with opposite spin orientation that interact via the attractive screened Coulomb potential −W(34). Unbound states consisting of independent electrons and holes can be interpreted as Stoner excitations, while low-energy bound states give rise to spin-wave modes.

Up to this point, the only simplification that was employed is the GW approximation for the electronic self-energy in Equation (35). In principle, the derivation mandates that the resulting expression for the spin susceptibility is evaluated with the self-consistent Green function that enters the self-energy and simultaneously solves the equation of motion (23)). However, such fully self-consistent GW calculations are overly expensive and, moreover, do not systematically improve upon standard perturbative approaches. In line with most applications of the GW approximation in materials simulations, the spin susceptibility is hence commonly evaluated with the Kohn–Sham Green function

(42) $$G_{\alpha }\left(r,r^{\prime };\omega \right)=\lim_{\eta \to 0}\left(\sum_{n}^{\mathrm{occ}.}\frac{\varphi_{n\alpha }\left(r\right)\varphi_{n\alpha }^{*}\left(r^{\prime }\right)}{\omega -\epsilon_{n\alpha }-i\eta }+\sum_{n}^{\mathrm{unocc}.}\frac{\varphi_{n\alpha }\left(r\right)\varphi_{n\alpha }^{*}\left(r^{\prime }\right)}{\omega -\epsilon_{n\alpha }+i\eta }\right)$$

using the single-particle wave functions and eigenvalues from Equation (18). Additionally, numerous additional approximations are typically employed in order to reduce the computational cost. In particular, the dynamically screened Coulomb potential $W(r_{3},r_{4};t_{3}-t_{4})$ in Equation (41) is almost always replaced by an instantaneous interaction $W\left(r_{3},r_{4}\right)\delta \left(t_{3}-t_{4}\right)$, which means that the frequency dependence of its Fourier transform is ignored [26]. As a further simplification, the spatial range of $W(r_{3},r_{4})$ is often cut off drastically, so that in an atom-centered basis, only matrix elements with four orbitals centered at the same atomic site are considered, for example in a basis of muffin-tin orbitals [42] or Wannier functions [43].

One notable consequence of these manifold approximations is that actual quantitative calculations almost always exhibit a violation of the Goldstone condition. The Goldstone theorem states that, in the absence of spin–orbit coupling or other symmetry-breaking effects, there exists a spin-wave mode at the Γ point in the Brillouin zone whose excitation energy corresponds to the Zeeman energy of the system [44]. Without external magnetic fields, this implies a gapless dispersion for magnetically isotropic materials like cobalt, iron or nickel. The violation of the Goldstone condition observed in practice is, on the one hand, caused by numerical effects, such as truncated basis sets, which lead to underconvergence. On the other hand, it stems from the discrepancy between the Kohn–Sham Green function in Equation (42) and the GW approximation, as well as the simplified static interaction *W* typically employed in Equation (41). Various correction schemes exist for the Goldstone error, which can be quite severe [45]. These range from modifying the ground-state band structure in various ways, such as a suitable adjustment of the exchange splitting [43,45], to a modification of the screened interaction, for example by a linear scaling factor [27,43].

In principle, as long as the exchange-correlation functional as well as the convergence parameters are chosen consistently in the self-consistent ground-state calculations and the subsequent linear-response calculations, a Goldstone error should not occur in TDDFT. In practice, however, the dynamical calculation often needs much higher convergence parameters. Applying these otherwise unnecessarily high convergence parameters to the ground-state calculations simply for the sake of consistency, while not unheard of [35], increases the computational cost enormously, which is why TDDFT works usually also employ Goldstone correction schemes. Correction schemes include a rigid shift of the entire dispersion [29,32], modifications of the Kohn–Sham susceptibility [31], or a linear scaling of the kernel [46].

Most correction schemes have in common that they exploit the knowledge of where the dispersion is supposed to begin according to the Goldstone theorem, running somewhat counter to the idea of ab initio. The reasoning for these schemes is thus of interest. First, a rigid shift of the dispersion has no direct physical justification but is the simplest option for a small Goldstone error. The same can be said for simply scaling or adjusting the exchange-correlation kernel, the screened interaction, or the Kohn–Sham susceptibility. Small adjustments here can be seen as numerical details, while a large quantitative alteration is difficult to justify. Lastly, modifications of the band structure, such as a manual adjustment of the exchange splitting by means of a scissor operator, are based on the argument that static Kohn–Sham DFT as well as the Green function in MBPT should reproduce key observables like the magnetization density exactly. The latter depends on the exchange splitting, which also has a direct influence on the spin-wave dispersion. If the initial static calculation yields an incorrect exchange splitting, then a manual adjustment, although not strictly ab initio, can be interpreted as a valid correction scheme to make up for deficiencies in the underlying approximate functionals.

## 3. Results and Discussion

In order to assess the performance of these methods, a comparison of results from different implementations with experimental data seems appropriate. In this section, we have, to the best of our knowledge, compiled an exhaustive list of works that utilize TDDFT or MBPT to calculate spin-wave dispersions. We concentrate on cobalt, iron and nickel, because the majority of ab initio works reported results for these systems. Additionally, they are often regarded as benchmarks and commonly used to test new computational schemes. We conclude the section with a short overview of applications to other materials, however.

### 3.1. Cobalt (fcc)

The most common experimental technique to measure spin-wave energies is neutron scattering. The magnetic moment of the neutron couples to the magnetization of the sample. This interaction can lead to a transfer of angular momentum and thereby to the excitation or annihilation of magnons. The dispersion is obtained from the energy and momentum loss of the incident neutrons. Neutron scattering experiments are available for many magnetic materials, but the highest detectable magnon energies are normally around 100 meV, depending on the neutron source and the monochromator, which effectively limits measurements to low-energy modes in the center of the Brillouin zone. For cobalt, however, Balashov [47] used a combination of scanning tunneling microscopy to excite magnons by electron scattering and inelastic scanning tunneling spectroscopy to determine their energies. The results, which are here used for comparison with the theoretical calculations, agree with neutron scattering measurements near Γ but cover the entire Brillouin zone.

**Figure 1:**
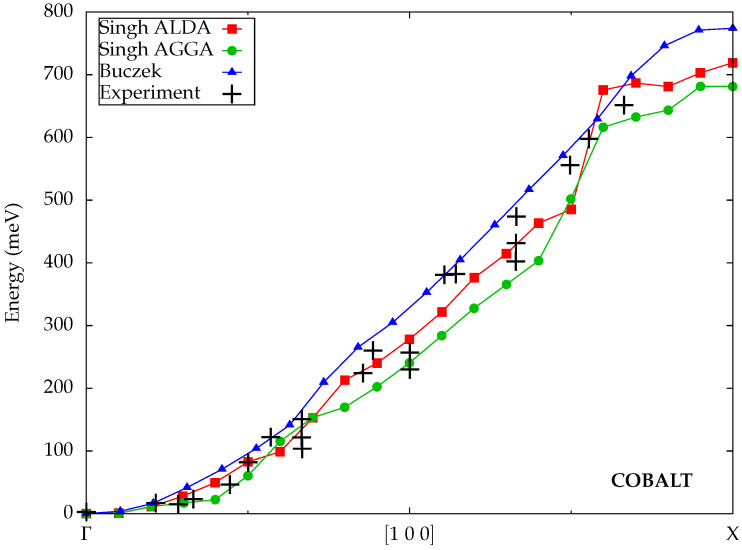
Calculated spin-wave dispersions for cobalt from Singh et al. [32] and Buczek et al. [49] (all TDDFT) compared to experimental data from Balashov [47].

Singh et al. [32] utilized the ELK code [48] to calculate spin-wave dispersions within TDDFT. It is based on the full-potential linearized augmented plane wave (FLAPW) scheme, which enables an efficient description of magnetic transition metals with localized *d* orbitals. As a unique feature, they also derived an expression for the exchange-correlation kernel in the adiabatic generalized gradient approximation, which they applied to several materials alongside the ALDA. The Goldstone condition is fulfilled via a rigid shift of the dispersion. The results for cobalt are compared to experimental data in Figure 1. One can observe that both ALDA and AGGA are in good agreement with the measurements. The AGGA tends to predict slightly lower spin-wave energies than the ALDA in this case, but the results do not give a clear indication which of the two yields better values. We will come back to this point later in this section.

Buczek et al. were early pioneers of ab initio spin-wave calculations within TDDFT, which they subsequently applied to a variety of bulk and thin-film systems [30,49,50,51,52,53,54]. They developed their own implementation based on the Korringa–Kohn–Rostoker (KKR) Green function method, which is described in detail in [50]. The exchange-correlation kernel was evaluated within the ALDA, as is standard in most TDDFT calculations. For compliance with the Goldstone condition, the exchange-correlation kernel is explicitly modified to yield a gapless dispersion; see [49] for details. Their results for cobalt in Figure 1 are in good agreement with the experimental data, albeit at slightly higher energies than those reported by Singh et al. at the same ALDA level.

**Figure 2:**
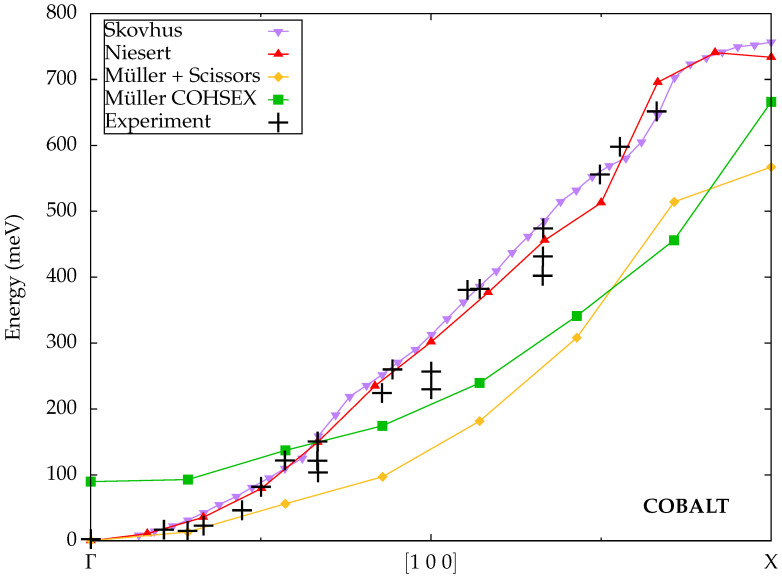
Calculated spin-wave dispersions for cobalt from Skovhus et al. [33], Niesert [29] (both TDDFT) and Müller et al. [45] (MBPT) compared to experimental data from Balashov [47].

Skovhus et al. [33] implemented spin-wave calculations within the existing linear-response TDDFT module of the GPAW code [55], which uses a basis of projector-augmented waves (PAWs), while the exchange-correlation kernel was evaluated within the ALDA. They shifted the calculated dispersion to fulfill the Goldstone condition. Their results for cobalt, shown in Figure 2, are once more in good quantitative agreement with the experimental data.

Niesert [29] performed spin-wave calculations for several materials using TDDFT within the ALDA. The ground-state DFT calculations were carried out with the FLEUR code [56], which relies on the FLAPW method. The subsequent construction of the transverse dynamic spin susceptibility made use of a mixed product basis [57]. The dispersion is shifted to comply with the Goldstone theorem. The calculated spin-wave dispersion for cobalt in Figure 2 is in good agreement with experiment and notably close to the results of Skovhus et al.

Müller et al. [45] explored MBPT for spin-wave calculations with a focus on different schemes to correct the violation of the Goldstone condition. The ground-state DFT calculations needed to construct the Kohn–Sham Green function were performed with the FLEUR code [56], whereas the dynamic transverse spin susceptibility was again determined in the mixed product basis [58]. The first correction scheme manipulates the exchange splitting between the majority and minority spin channels, whose size influences the energy gap of the spin-wave dispersion at the Γ point. By applying a scissor operator to shift the two spin channels relative to one another, the exchange splitting can thus be tuned until the gap vanishes and the Goldstone condition is satisfied. This modification of the Kohn–Sham Green function can be seen as a pseudo-renormalization, which mimics the missing renormalization of the Green function in the GW approximation to some extent but leaves the screened interaction *W* unaffected. The second scheme renormalizes the Kohn–Sham Green function explicitly with a static version of the GW self-energy, known as the Coulomb-hole screened-exchange (COHSEX) method, which is consistent with replacing the dynamically screened interaction *W* in Equation (41) by its static limit. As illustrated in Figure 2, however, it fails to correct the Goldstone error completely, because there is no continuous adjustable parameter in this scheme and various additional numerical approximations also influence the results. Furthermore, the dispersion is significantly too flat in both schemes compared to the experimental data as well as equivalent results from TDDFT calculations.

Overall, we observe that cobalt does not appear to be an overly problematic material for spin-wave calculations. With the exception of Müller et al. [45], all reported theoretical results agree well with the experimental data [47], and there is little deviation between the different independent implementations of TDDFT and ALDA.

### 3.2. Iron (bcc)

For iron, we compare with two frequently cited sets of experimental data, one obtained by Collins et al. [59] in 1969 at room temperature and the other by Loong et al. [60] in 1984 at 10 K. Due to the technical constraints of neutron scattering, the measurements only cover low-energy spin-wave excitations up to about 150 meV near Γ in the center of the Brillouin zone. Their deviation allows an estimate of how the experimental values may depend on the measurement techniques, sample quality or environmental conditions, and illustrates that their role as a quantitative benchmark must be taken with some caution. In light of the more recent measurement by Loong et al. as well as the experimental details, such as the low temperature that corresponds better to the setup of most theoretical simulations, the latter might seem preferable, but a final judgment on the accuracy cannot be made without further independent confirmation. One important message is that theoretical researchers must be aware of this spread and take care not to focus on just a single dataset that fits their own numerical results best.

**Figure 3:**
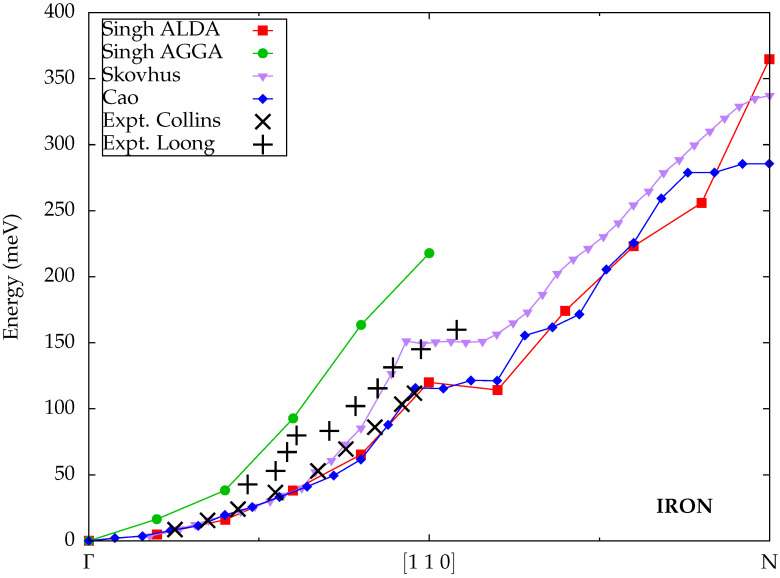
Calculated spin-wave dispersions for iron from Singh et al. [32], Skovhus et al. [33] and Cao et al. [35] (all TDDFT) compared to experimental data from Collins et al. [59] and Loong et al. [60].

Unlike for cobalt, the results from Singh et al. [32] for iron in Figure 3 exhibit significant deviations between ALDA and AGGA, as the latter yields substantially higher spin-wave energies. The ALDA is in excellent agreement with the experimental data from Collins et al., while those from Loong et al. lie between the two theoretical curves. Thus, the ALDA appears to be in better overall agreement with the experimental data, and its substitution by the more elaborate AGGA kernel does not lead to a systematic improvement. The AGGA however seems to better capture the dampening of the spin waves, as no clear excitations can be extracted from the theoretical calculations past the point where the available experimental data ends.

The spin-wave dispersion for iron reported by Skovhus et al. [33] within the ALDA is in equally good agreement with the experimental data from Collins et al. at small wave vectors near the Γ point. For larger wave vectors, the calculated spin-wave energies increase more steeply, however, and are closer to the larger experimental values from Loong et al. Nevertheless, the overall shape of the dispersion is very similar to the ALDA results from Singh et al. and exhibits the same kink halfway along the Γ–N path. Unfortunately, both experimental datasets terminate at this point and cannot confirm this peculiar feature.

Cao et al. [35] calculated spin-wave dispersions, based on plane waves and pseudopotentials, by solving the Sternheimer equation within time-dependent density-functional perturbation theory, which they implemented into the Quantum ESPRESSO code [61,62]. The ALDA was used for the exchange-correlation kernel. They fulfilled the Goldstone condition by using consistent convergence parameters between the ground-state calculations and the dynamical calculations. As shown in Figure 3, their results are very similar to those from Singh et al. and in close agreement with the experimental data from Collins et al.

**Figure 4:**
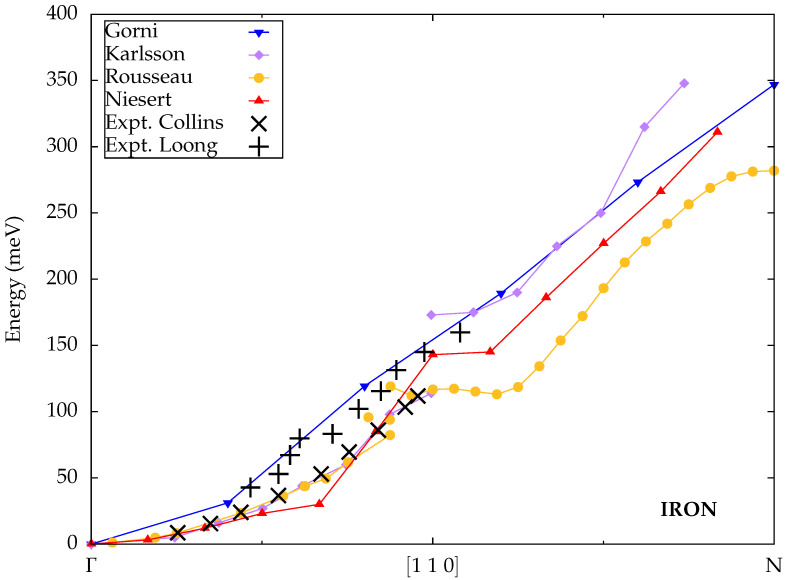
Calculated spin-wave dispersions for iron from Gorni et al. [36], Rousseau et al. [31], Niesert [29] (all TDDFT) and Karlsson et al. [42] (MBPT) compared to experimental data from Collins et al. [59] and Loong et al. [60].

Gorni et al. [36] used the Liouville–Lanczos approach [63] as an alternative solver for time-dependent density-functional perturbation theory, also within Quantum ESPRESSO. Their implementation has since been released as a Quantum ESPRESSO component under the name turboMagnon [64]. The Liouville–Lanczos approach has the advantage that the computationally most expensive steps are decoupled from the excitation frequency and must hence only be carried out once for each wave vector. The dynamical calculations are not decoupled from the ground state calculations for this method; hence, consistency in the convergence parameters is enforced, fulfilling the Goldstone condition. The frequency dependence of the transverse spin susceptibility can afterwards be efficiently evaluated. As usual, the ALDA is chosen for the exchange-correlation kernel. The results shown in Figure 4, located at slightly higher energies than those from most other implementations, are in agreement with the experimental data from Loong et al. The small number of wave vectors considered in [36] is not sufficient to resolve the kink observed in other theoretical calculations.

Karlsson and Aryasetiawan pioneered the MBPT formalism for spin waves in a full ab initio context [26] and demonstrated its practical feasibility for selected materials, including iron [42]. The implementation was based on the linear muffin-tin orbital (LMTO) method. To reduce the computational cost, the screened interaction in Equation (41) was assumed to be static, and only matrix elements with all muffin-tin orbitals centered at the same atomic site were considered, thus limiting the spatial range to an effective Hubbard-like onsite interaction. The results initially follow the experimental data from Collins et al. but exhibit a substantial discontinuity in the region where the experimental dataset ends. At larger wave vectors, the dispersion reappears at higher energies, similar to Gorni et al. and in an apparent extrapolation of the experimental values from Loong et al. Such a discontinuity in the spin-wave dispersion has, to the best of our knowledge, not been directly observed in experimental measurements but was predicted by Ododo et al. [65] based on an analysis of experimental data for the temperature-dependent magnetization of iron.

In their ALDA calculations, Rousseau et al. [31] put the focus on the violation of the Goldstone condition and possible correction schemes. After discussing common approaches to this problem, such as a rigid energy shift or a simple scaling of the kernel, they proposed a small additive correction to the Kohn–Sham spin susceptibility constructed in such a way that the magnetization density obtained from the Kohn–Sham susceptibility is identical to the self-consistent result from the ground-state DFT calculation. The latter is carried out with Quantum ESPRESSO in combination with a Wannier-function representation of the susceptibility. The calculated spin-wave energies for iron closely follow the measured data from Collins et al. for small wave vectors, obeying the Goldstone condition as intended. Similar to Karlsson and Aryasetiawan, there is a discontinuity near the point where the experimental dataset ends, although it is much smaller here.

Niesert [29] also reported spin-wave energies for iron within the ALDA. The predicted dispersion appears slightly too flat for small wave vectors and suggests a discontinuity, although this is not explicitly analyzed. For large wave vectors, the results are again similar to those from other implementations.

**Figure 5:**
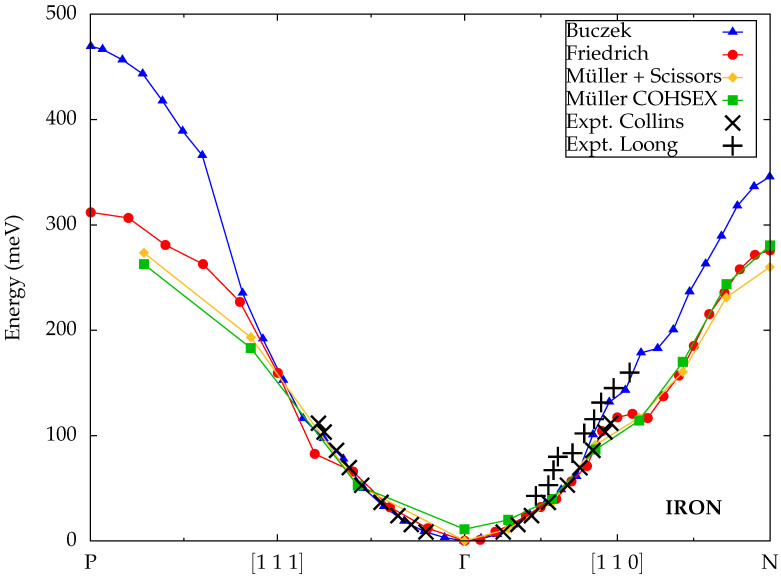
Calculated spin-wave dispersions for iron from Buczek et al. [49] (TDDFT), Friedrich et al. [27] and Müller et al. [45] (both MBPT) compared to experimental data from Collins et al. [59] and Loong et al. [60].

While most theoretical studies only calculated spin-wave energies for iron along the [110] direction, a few extended the path in the Brillouin zone to the [111] direction, for which experimental measurements are also available. The dispersion obtained by Buczek et al. [49] within the ALDA, displayed in Figure 5, shows good agreement with the experiments from Collins et al., although for larger wave vectors along the Γ–N direction, the calculated energies are higher and closer to the data from Loong et al.

Friedrich et al. [27] and Müller et al. [45] used the same implementation for spin-wave calculations within MBPT based on the FLAPW method, initially developed by Şaşıoğlu et al. [43]. The ground-state DFT calculations were performed with the FLEUR code [56]. The subsequent construction of the spin susceptibility employed a mixed product basis in combination with a projection onto maximally localized Wannier functions, implemented as part of the SPEX code [66]. However, the Goldstone error correction was treated in different ways. While Friedrich et al. [27] scaled the onsite matrix elements of the static screened Coulomb interaction in the Wannier basis to obtain a vanishing energy gap, Müller et al. [45] applied either a scissor operator to tune the exchange splitting or a renormalization with the nonlocal but static COHSEX self-energy as discussed above for cobalt. The results for iron are displayed in Figure 5. Despite the different correction schemes, they are remarkably close not only to each other but also to the experimental data by Collins et al., although the COHSEX scheme again exhibits a remaining finite Goldstone error. Furthermore, it is notable that the calculated spin-wave energies are very similar to the ALDA results from Buczek et al. around the Γ point but substantially lower near the edges of the Brillouin zone, especially along the Γ–P direction.

### 3.3. Nickel (fcc)

Experimental measurements for nickel were reported by Lynn et al. [67] for small wave vectors close to Γ and by Mook et al. [25] for intermediate wave vectors up to a maximum magnon energy around 200 meV. Although nickel is an elemental ferromagnet like cobalt or iron, the spin-wave dispersion has a more complex structure, as the experimental data show two clearly distinct branches along the [100] direction, sometimes referred to as acoustic and optical branches in analogy to phonons. This makes nickel arguably the most interesting of the three materials and a particular challenge for theoretical simulation methods.

Although the coexistence of two separate magnon branches in nickel is experimentally well established [25], its physical interpretation remains an open question. In contrast to phonons, where the discrete nature of the crystal lattice admits only a finite number of degrees of freedom per unit cell for the atomic motion, spin waves arise from excitations of the electron system, whose spatial density distribution and magnetization are continuous with an infinite number of degrees of freedom. Therefore, multiple magnon branches may occur even in materials with one atom per unit cell and do not require a multiatom basis or Brillouin-zone folding. The actual manifestation depends on the details of the electronic structure, however. In the case of nickel, theoretical simulations with a proper material-specific band structure indeed predict two distinct branches along the [100] direction [68], but while the magnon energies are easily obtained from the double-peak structure of the imaginary part of the transverse dynamic spin susceptibility, the nature of the induced spin dynamics remains unclear. Another open question is why double branches have not been experimentally detected in other directions. As the measurements by Mook et al. [25] had a lower resolution for the [111] than for the [100] direction, it was suggested that the splitting may not have been observable. Indeed, calculations by Karlsson and Aryasetiawan [42] indicate that a larger broadening leads to a merger of the two distinct resonances, giving the impression of a single peak. Nevertheless, a comprehensive theoretical and experimental explanation is still required.

**Figure 6:**
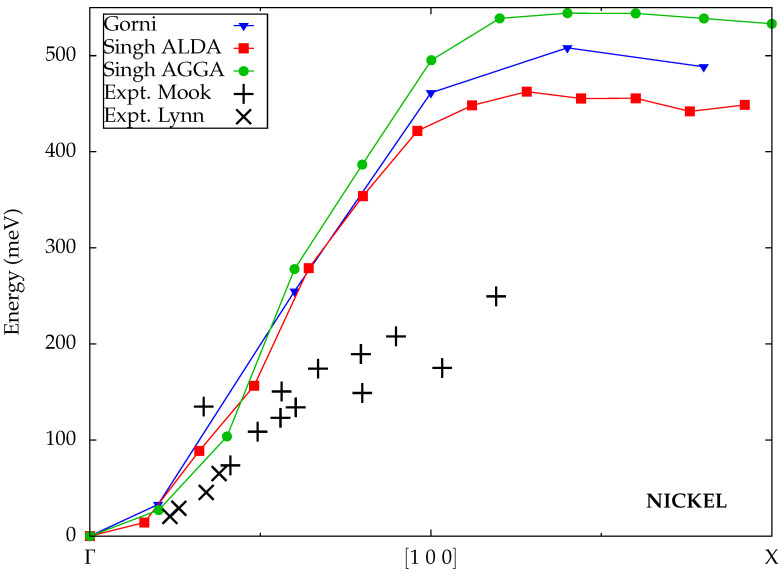
Calculated spin-wave dispersions for nickel from Gorni et al. [36] and Singh et al. [32] (all TDDFT) compared to experimental data from Mook et al. [25] (plus symbols) and Lynn et al. [67] (cross symbols).

The results from Gorni et al. [36], calculated within the ALDA and displayed in Figure 6, overestimate the experimentally measured spin-wave energies in nickel considerably. Moreover, the obtained dispersion exhibits only a single branch, which is much too steep in comparison with the experimental data.

The ALDA results from Singh et al. [32] are close to those from Gorni et al. The AGGA yields very similar spin-wave energies as the ALDA for small wave vectors, but for larger wave vectors near the edge of the Brillouin zone, they are significantly higher, leading to an even larger deviation from the experimental values. Singh et al. attributed the poor performance of the AGGA to the exchange splitting in the GGA band structure, which is even more overestimated than it already is in the LDA for nickel. Evidently, the AGGA kernel does not improve on the ALDA kernel’s failure to correct the discrepancy between the Kohn–Sham eigenvalues and the true quasiparticle band structure.

**Figure 7:**
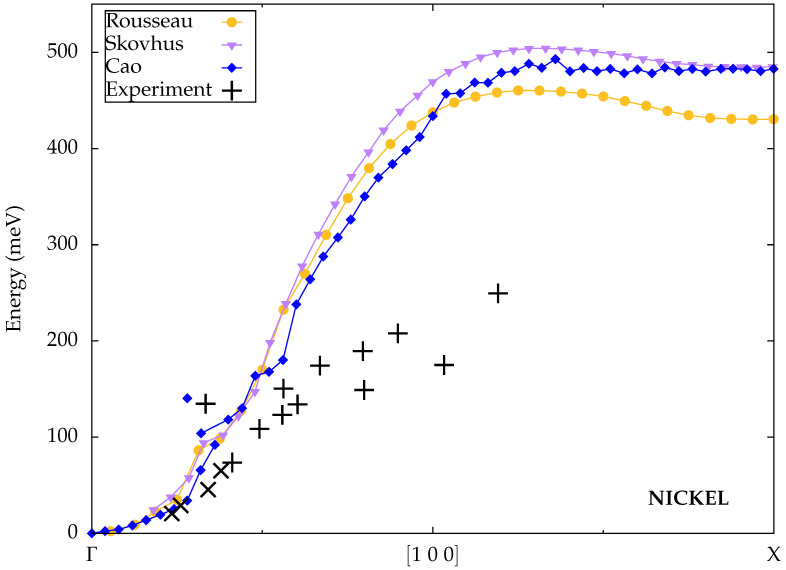
Calculated spin-wave dispersions for nickel from Rousseau et al. [31], Skovhus et al. [33] and Cao et al. [35] (all TDDFT) compared to experimental data from Mook et al. [25] (plus symbols) and Lynn et al. [67] (cross symbols).

Rousseau et al. [31] also performed spin-wave calculations for nickel within the ALDA. Their results are displayed in Figure 7. The dispersion contains only one branch, although a minor kink is visible at small wave vectors about 20% along the Γ–X path. The calculated magnon energies are compatible with the first data points from Lynn et al. for the smallest wave vectors, but the dispersion quickly becomes too steep and again overestimates the experimental measurements substantially at large wave vectors.

The results from Skovhus et al. [33] are very similar to those from Rousseau et al., with a single branch and the same kink at small wave vectors, although at large wave vectors, the calculated magnon energies are somewhat higher.

Cao et al. [35] reported spin-wave energies for nickel that are, overall, quite similar to the ALDA results from Rousseau et al. and Skovhus et al., but they observed a double-peak feature in the spectral function that they interpreted as the onset of a second magnon branch instead of a kink, as indicated in Figure 7. However, the presence of two distinct branches occurs only in a narrow interval of very small wave vectors, which is not consistent with the experimental measurements by Mook et al. who found an upper and lower branch throughout a large part of the Brillouin zone along the [100] direction.

**Figure 8:**
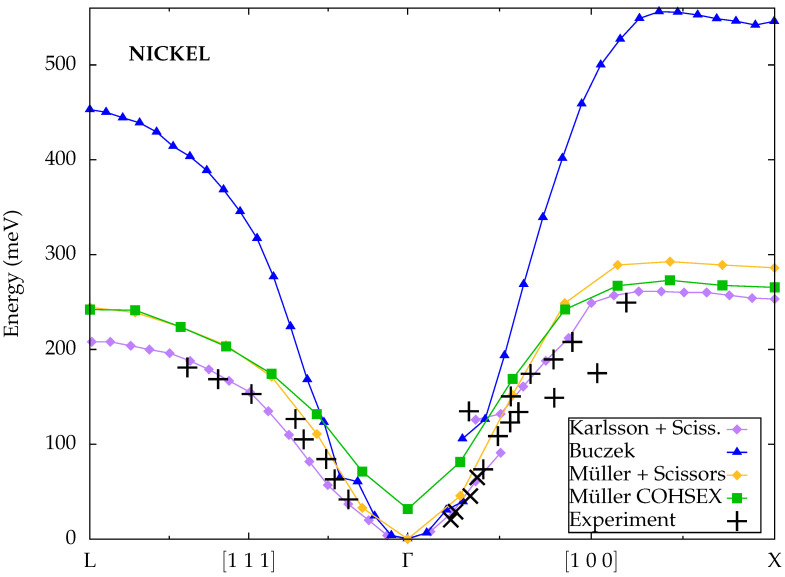
Calculated spin-wave dispersions for nickel from Karlsson et al. [42] (MBPT), Buczek et al. [49] (TDDFT) and Müller et al. [45] (MBPT) compared to experimental data from Mook et al. [25] (plus symbols) and Lynn et al. [67] (cross symbols).

Buczek et al. [49] calculated spin-wave energies for nickel not only along the [100] direction, but also along the [111] direction, for which the experimental data from Mook et al. indicate just a single branch. The results, displayed in Figure 8, are in good agreement with other calculations based on ALDA and likewise overestimate the experimental values substantially at large wave vectors. Although they observed the onset of a second magnon branch along the [100] direction, the original lower branch vanishes around the same wave vector where the new upper branch appears, resulting in an effective discontinuity.

Karlsson and Aryasetiawan [42] based their calculations for nickel on MBPT. With the unmodified Kohn–Sham Green function and a static screened onsite Coulomb interaction scaled to fulfill the Goldstone theorem, the dispersion turns out similar to the ALDA. The results displayed in Figure 8 were instead obtained after manually reducing the exchange splitting from 0.6 eV in the LDA to the experimental value of 0.3 eV, which lowers the energy scale of spin-wave excitations and brings the results into good agreement with the experimental measurements. Along the [100] direction, a second branch is clearly observed as double peaks in the calculated susceptibility, but similarly to Cao et al., the lower branch vanishes shortly after the onset of the upper branch, and there is only a small overlap region where both branches occur simultaneously.

Müller et al. [45] also used MBPT together with a scissor operator, but the exchange splitting was optimized to enforce the Goldstone condition in this case. The resulting value turns out similar to the experimental number, however, and the calculated magnon energies are hence similar to those from Karlsson and Aryasetiawan, albeit slightly higher at large wave vectors. The COHSEX self-energy renormalization also modifies the electronic band structures and the exchange splitting, but in a nonempirical way without adjustable parameters. While this leads to decent overall agreement with the experimental measurements, the Goldstone theorem is again not strictly fulfilled, since a finite energy gap remains at Γ. Both schemes yield only a single magnon branch, although the coarse grid of wave vectors may simply be insufficient to resolve the overlap region between separate branches.

**Figure 9:**
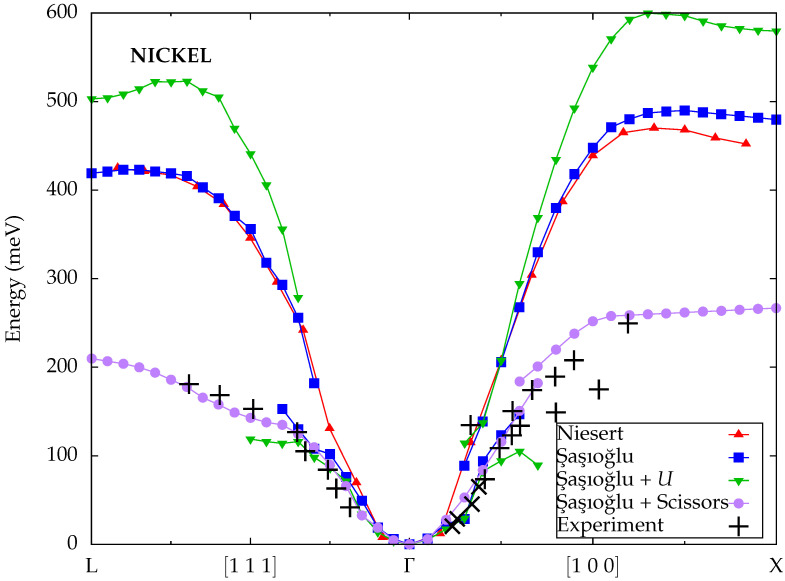
Calculated spin-wave dispersions for nickel from Niesert [29] (TDDFT) and Şaşıoğlu et al. [43] compared to experimental data from Mook et al. [25] (plus symbols) and Lynn et al. [67] (cross symbols).

The results from Niesert [29], which are shown in Figure 9, follow those from other ALDA implementations. In particular, there is only a single magnon branch that rises to much higher energies at large wave vectors than experimentally measured.

Şaşıoğlu et al. [43] explored different approaches for spin-wave excitations within MBPT. The ground-state calculations were based on the FLAPW method and performed with the FLEUR code [56]. In addition to the standard LDA exchange-correlation functional, they also tested LDA+*U* with an additional Hubbard *U* term to describe the strong onsite correlation of the localized electrons in the partially filled 3*d* orbitals of nickel. The dynamic spin susceptibility was constructed using a mixed product basis and projection onto Wannier functions, as implemented in the SPEX code [66]. In addition to Kohn–Sham Green functions based on LDA and LDA+*U* band structures, they also considered a scissor operator that reduced the exchange splitting to the experimental value of 0.3 eV in analogy to Karlsson and Aryasetiawan. In all cases, the Goldstone condition was enforced by a linear scaling of the onsite matrix elements of the static screened Coulomb interaction in the Wannier basis. The scaling factor is largest for the LDA+*U* scheme at 1.8 and smallest with the scissor operator at 1.1, with the value 1.5 for the LDA in between. All three approaches predict two magnon branches with a small overlap region along the [100] direction, but the energy scales differ significantly. The unmodified LDA results are quite similar to those from ALDA calculations within TDDFT, while LDA+*U* yields even higher energies. Only the dispersion obtained with the scissor operator is in decent quantitative agreement with the experimental data. Incidentally, Şaşıoğlu et al. also found double branches or a kink along the [111] direction, which had not been reported previously and is not confirmed by the experimental data, although it is notable that the measurements from Mook et al. also suggest a marked change in the slope of the dispersion at this point.

### 3.4. Other Materials

In the following we present a brief overview of applications of spin-wave excitations in various materials other than the elemental transition metals already covered above. First of all, however, we mention a few relevant works that, for various reasons, were not included in the previous comparison with experiments for cobalt, iron and nickel.

Some of the earliest ab initio spin-wave calculations were performed in the 1980s by Cooke et al. [68,69,70] for iron and nickel and by Callaway et al. [71] for nickel. Although the computational approach is consistent with the scope of this review, severe approximations were made both for the electronic band structure and the treatment of dynamical correlation, which are not on par with modern implementations. While the reported agreement with experiments is decent, this should hence be taken with caution.

The first work that truly applied TDDFT in the modern sense to magnetic excitations is by Savrasov [34], who solved the Sternheimer equation within time-dependent density-functional perturbation theory to calculate the spin-wave dispersions of iron, nickel and chromium. The results are in good quantitative agreement with experimental data for iron. For nickel, there is an upper second magnon branch, albeit at too-high energies.

Okumura et al. [72] reported an implementation of MBPT for spin-wave calculations based on Green functions from the quasiparticle self-consistent GW method [73,74]. Their results for iron and nickel are in good agreement with experiments, but it is unclear from the published data whether there are one or two magnon branches for nickel.

#### 3.4.1. Heusler Alloys

Foremost among applications to other classes of materials are the Heusler and half-Heusler alloys, a group of intermetallic compounds with complex and interesting magnetic properties. Spin-wave calculations have been reported for NiMnSb [32,50], Co_2_MnSi [32,50], Cu_2_MnAl [50,75,76], CrMnSb [77], Ni_2_MnSn [76] and Pd_2_MnSn [76]. Buczek [50] generally achieved good agreement with available experiments for small wave vectors within the ALDA while overestimating the excitation energies for larger wave vectors. Singh et al. [32] obtained very similar results to Buczek within the ALDA, whereas the AGGA vastly overestimated the spin-wave energies. The results from Liu et al. [75], obtained with time-dependent density-functional perturbation theory, agree with Buczek. Lastly, Okumura et al. [76] achieved good agreement with experimental measurements based on MBPT with the quasiparticle self-consistent GW method.

#### 3.4.2. Bulk Transition Metals and Their Binary Compounds

Further bulk transition metal compounds for which ab initio spin-wave calculations were reported are hcp Co [33,50,72], bcc Co [29], fcc Fe [29,38], bcc Ni [29], CrO_2_ [27,75], FeCo [72,78], FeRh in the ferromagnetic and antiferromagnetic phases [79], MnBi [80], Cr_2_O_3_ [46], Cr [34,46] and FeSn [81]. Although the MBPT implementation from Okumura et al. [72] yielded good agreement with experiments for elemental ferromagnets and Heusler alloys, the results for spin waves in FeCo deviated from experimental data and exhibited a significantly flatter dispersion. Skovhus et al. [46,80] employed the LDA+*U* scheme in TDDFT similar to Şaşıoğlu et al. [43] in MBPT. The ground-state calculations were performed with a fixed Hubbard *U*, and the ALDA kernel was subsequently scaled to satisfy the Goldstone condition. With this modification, the agreement with experiments was improved in comparison to standard ALDA calculations. Buczek et al. [50], Skovhus et al. [33] and Friedrich et al. [27], with their previously discussed implementations, achieved good agreement with experiments for hcp Co and CrO_2_, while the calculated spin-wave dispersion from Liu et al. [75] for CrO_2_ was slightly flatter than experimentally observed. Sandratskii et al. [79] and Zhang et al. [81] performed ALDA calculations for FeRh and FeSn, respectively, both in decent agreement with experimental data.

#### 3.4.3. Transition Metal Thin Films

Finally, spin-wave calculations were also performed for low-dimensional systems, which are of great importance for technological applications. The earliest applications to two-dimensional layers were reported by Tang et al. [82] and by Muniz et al. [83] for thin films of iron, both free-standing and adsorbed on a tungsten substrate. Although their calculations are based on TDDFT, they incorporate heavy simplifications, such as band structures obtained from an empirical tight-binding scheme. Later, Buczek et al. [30,49,53] used their ab initio ALDA implementation to investigate a variety of thin-film configurations. These include thin films of iron adsorbed on copper and tungsten, two and three monolayers of iron on tungsten and copper, respectively, as well as thin films of cobalt and nickel adsorbed on copper. Where experimental data is available for comparison, the agreement is decent.

## 4. Conclusions

In this review, we set out to give an overview of the theoretical foundations of modern ab initio spin-wave calculations and their applications to magnetic materials. We focused on time-dependent density-functional theory and many-body perturbation theory, the two available methods that allow, in principle, an exact computation of spin-wave energies in solids. The examples highlighted in the previous section demonstrate that great strides were indeed made with respect to the numerical implementation during the last few decades, and that a variety of codes using different basis sets and computational strategies have been successfully demonstrated. So far, most computer codes are still private development versions, with the notable exception of turboMagnon [64], which has been released as a component of the Quantum ESPRESSO distribution. As the available computational power continues to increase, the need for major numerical simplifications is expected to diminish further, and convergence will become less of an issue. From heavily simplified electronic band structures and model interaction potentials in early works to DFT or even self-energy-corrected band structures and ab initio screening of the Coulomb interaction in more recent works, the calculations have already become more and more realistic. An assessment of the present state of the art is thus expedient to guide further development efforts.

One reassuring observation from this survey is that all reported spin-wave calculations using TDDFT are in good quantitative agreement, although the implementations rely on very different basis sets and computational approaches, such as spectral summation, time-dependent density-functional perturbation theory with the Sternheimer equation, or the Liouville–Lanczos method. Due to the manageable computational expense, the calculations can be well converged to eliminate basis-set-related errors; however, the good intrinsic compliance with the Goldstone condition limits the influence of alternative correction schemes. On the other hand, the ALDA, which is almost universally employed, constitutes a major approximation, and the calculated spin-wave energies may deviate significantly from experiments, notably for nickel. In principle, the exchange-correlation kernel serves two purposes: it must correct the discrepancy between the Kohn–Sham eigenvalues of static DFT and the true quasiparticle band structure, and it must account for dynamic exchange and correlation effects of spin-flip electron–hole excitations, but the ALDA performs poorly in the first task [84]. As a consequence, large deviations occur in materials like nickel where the exchange splitting of the Kohn–Sham eigenvalues does not match the experimental value. Additionally, the simplified static and local approximation of the kernel within the ALDA appears unable to reproduce intricate features like the experimentally observed occurrence of two distinct magnon branches along the [100] direction of nickel. Therefore, the quest for exchange-correlation kernels with a more realistic wave vector and frequency dependence remains an important task. The only viable alternative to the ALDA that has been tested so far, the AGGA, does not lead to systematic improvements [32]; instead, the deviation from experimental measurements tends to increase. Different strategies are hence needed.

Existing implementations of MBPT for magnetic excitations also share a number of common traits, such as the time-dependent GW approximation for the interaction kernel and the replacement of the dynamically screened Coulomb interaction by its static limit in Equation (41). However, the numerical operations are more complex than in TDDFT and make fully converged calculations much more challenging. In combination with an effective spatial cutoff by including only the onsite matrix elements of the screened interaction in a particular representation, such as LMTO or Wannier functions, this makes the results more sensitive to the choice of basis sets and other computational parameters. Inconsistencies between various theoretical and numerical simplifications further lead to a large Goldstone error, which make the results dependent on the specific correction scheme. Consequently, the reported spin-wave energies vary more widely than in TDDFT. It is not surprising that evaluating the spin susceptibility with the Kohn–Sham Green function and using a local onsite Coulomb interaction, as is common, yields results that are very similar to the ALDA. According to the original derivation, the susceptibility should instead be evaluated with the properly renormalized GW Green function. Starting from a band structure with self-energy corrections in the COHSEX or quasiparticle self-consistent GW approximation, or just applying a simple scissor operator to adjust the exchange splitting, indeed leads to better agreement with experimental data, but there is presently no consistent scheme that satisfies the Goldstone condition reliably without adjustable parameters. Further research is hence desirable to resolve this issue in a satisfactory way. On the plus side, the degree of nonlocal and dynamic exchange-correlation effects in present implementations of MBPT is sufficient to predict features like the appearance of a second magnon branch in nickel in a correct qualitative manner.

Incidentally, on a very different note, this review also draws attention to the scarcity of reliable experimental benchmark data. Most existing experimental measurements are based on neutron scattering and cover only a small region of low spin-wave energies in the center of the Brillouin zone. Datasets obtained by different methods that extend over the entire Brillouin zone, such as the measurement by Balashov [47] for cobalt based on inelastic scanning tunneling spectroscopy, are rare and not available for all materials. Additionally, not all experimental data reported in the literature are mutually consistent, and the influence of different measurement techniques, sample preparation and ambient conditions, such as temperature, often remains unclear. To support the development of more refined theoretical simulation methods, new accurate experimental measurements of spin-wave dispersions in different magnetic materials would definitely be helpful.

## Data Availability

No new data were created or analyzed in this study. Data sharing is not applicable to this article.

## Abbreviations

The following abbreviations are used in this manuscript:

AGGA	adiabatic generalized gradient approximation
ALDA	adiabatic local-density approximation
COHSEX	Coulomb-hole screened-exchange
DFT	density-functional theory
FLAPW	full-potential linearized augmented plane wave
GGA	generalized gradient approximation
KKR	Korringa–Kohn–Rostoker
LDA	local-density approximation
LMTO	linear muffin-tin orbital
MBPT	many-body perturbation theory
PAW	projector augmented wave
TDDFT	time-dependent density-functional theory
